# Neuropsychological and Neuropsychiatric Concerns for Deep Brain Stimulation in Dystonia: Preoperative Profiles in a Deep Brain Stimulation Cohort and Postoperative Changes in Three Case Series Reports

**DOI:** 10.7759/cureus.3507

**Published:** 2018-10-29

**Authors:** Sophie J Zacharia, Giannis Sokratous, Mike Samuel, Angela Costello, Keyoumars Ashkan, Paul Shotbolt

**Affiliations:** 1 Psychology, King's College Hospital, London, GBR; 2 Neurosurgery, King's College Hospital, London, GBR; 3 Neurology, King's College Hospital, London, GBR; 4 Psychiatry, King's College Hospital, London, GBR; 5 Psychiatry, Institute of Psychiatry, Psychology and Neuroscience, London, GBR

**Keywords:** dbs, neuropsychology, neuropsychiatry

## Abstract

Cognitive deficits and psychiatric morbidities are commonly detected in dystonia. Psychiatric disturbances are of particular clinical concern as they not only contribute to poor quality of life and disease associated burden, but also exacerbate motor and cognitive symptoms. Bilateral deep brain stimulation of the globus pallidus internus improves motor symptoms in treatment-resistant dystonia, but its implications for non-motor manifestations are poorly understood. Improved prediction of cognitive and neuropsychiatric outcomes is important in deep brain stimulation (DBS) research and we aim to assess the latter through established assessment tools.

We document the cognitive and neuropsychiatric profiles in 11 primary and 10 secondary dystonia patients attending our DBS clinic. We performed routine multidisciplinary assessments including a comprehensive battery of neuropsychometric tests and detailed neuropsychiatric evaluations. Post-operative assessment outcomes are reported for three patients in case series.

The main cognitive deficit was on the Brixton test of spatial anticipation in primary dystonia. Background medical history included psychiatric illness in 38.1% of the patients with 76% of patients having mood abnormalities confirming elevated psychiatric morbidity in this population. Depressive illness was more prominent in primary, whereas clinically relevant histories in secondary dystonia were varied. Of the 21 patients three were able to perform on selected tests due to extensive limitations of their dystonia. No obvious alteration in intellectual functioning following DBS surgery relative to performance at the time of initial assessment was observed.

The frequency of individual impairments suggests that difficulties associated with dystonia are likely to be of clinical relevance to cognitive functions in the majority of patients. In particular, current findings suggest that executive difficulties related to inductive processes and spatial learning may be a common in primary dystonias. Psychiatric disturbances demand recognition as a central aspect of dystonia as they contribute to overall disease burden, poor quality of life and exacerbated motor disabilities. The available evidence provides overwhelming suggestion that vulnerability to depression is inherent to the dystonia phenotype.

## Introduction

Cognitive functions are frequently compromised in dystonia patients. However, the reported deficits are mild and there are inconsistencies in the literature; some studies suggest that executive functions, allocation of attention, fine motor speed and visuospatial functions are impaired in primary dystonia [1-3] but these findings have not been consistently replicated [4]. There is no indication of progressive cognitive decline in dystonia [5] and individual impairments are confounded by factors such as low-level intellectual functions, early age at dystonia onset, severe motor disability and maladaptive behaviors [6].

By contrast, various lines of evidence suggest that psychiatric disturbances are intrinsic to the neurobiology of dystonia. There is a high prevalence of psychiatric illness in dystonia and common diagnoses include depression, anxiety and obsessive-compulsive disorder. In addition, depressive illness does not correlate with the severity of dystonia and in many cases diagnoses precede the onset of dystonia by a number of years [7]. Therefore, distinct neuropsychiatric profile in dystonia may not be attributed to the effects of chronic motor disability in dystonia.

When motor symptoms are far advanced, pharmacological therapies are often unsatisfactory or limited by adverse effects [8]. Deep brain stimulation (DBS) is a reversible “non-lesioning” surgical intervention that is continuously applied via stereotactically implanted macroelectrodes to the globus pallidus internus (GPi) for the treatment of dystonia [9].

Motor improvement following DBS-GPi in dystonia is well established but complications can also occur [10]. Of particular concern, there is evidence to suggest an elevated prevalence of suicide in dystonia patients following DBS [11]. Suicides were reported up to nine years postoperatively; patients showed excellent motor responses to surgery and risk factors included severe depression and overly optimistic expectations of surgery. These findings underscore the need for thorough neuropsychiatric examination on preoperative assessment, management of patients’ expectations, and sustained post-operative surveillance and psychiatric support as part of the treatment procedure.

In this study, we have described neuropsychological and neuropsychiatric profiles in a specialized population of dystonic patients undergoing preoperative assessment for DBS before commenting on the potential mechanisms and functional relevance of these non-motor features in dystonia. In a separate sub-study, postoperative outcomes are reported for three patients. The potential mechanisms of non-motor features are considered in relation to DBS and the predictive value of routine pre-operative examination is discussed.

## Case presentation

Patients 

Group of 22 dystonia patients who were referred to our multidisciplinary DBS clinic. Indications for referral were motorically advanced dystonia disorders with medically intractable symptoms following neurological evaluation. The cohort was subcategorized according to classification of dystonia disorders: patients had been diagnosed with a primary dystonia (PD) (n = 11) or a secondary dystonia (SD) to some other cause (n = 11) (trauma, stroke, cerebral palsy or hypoxia at birth). Demographic and clinical characteristics for the entire cohort and dystonia patient subgroups are summarized in Table 1. Given that the relevance of recording handedness is to signify cerebral dominance, 15 patients were right-hand dominant but three of these patients had switched to left-hand use due to motor difficulties; seven patients were left-hand dominant, one of which had switched to right-hand use due to motor difficulties.

**Table 1:** Clinical characteristics of patients with primary or secondary dystonia. TBM: Tuberculosis meningitis; ICA: Internal carotid artery; MRA: Medial renal artery; LH: Left hemisphere; 1: Right; 0: Left

		Age	Sex	Handedness	Type of dystonia	Precipitant/presumed cause
Primary dystonia patients						
	P1.	49	M	1	Cervical	
	P2.	61	F	1	Cervical, blepharospasm	
	P3.	22	F	1	Primary generalized	
	P4.	60	F	0	Right torticollis	
	P5.	55	F	1	Cervical	Genetic, family history
	P6.	20	M	1	Spasmodic torticollis, myoclonus	Hypoxia at birth requiring resuscitation
	P7.	64	M	1	Primary generalized	
	P8.	43	M	1	Primary generalized, cervical	Genetic, family history
	P9.	55	F	1	Primary generalized	
	P10.	62	M	1	Spasmodic torticollis	Genetic
	P11.	48	M	1	Cervical	Genetic
Secondary dystonia patients						
	S1.	55	M	1	Tardive dystonia and dyskinesia, blepharospasm	Neuroleptic toxicity
	S2.	24	F	1	Athetoid dystonic cerebral palsy	Cerebral palsy
	S3.	23	M	1	Secondary generalized, pallidal necrosis	Pallidal necrosis, Leigh’s disease
	S4.	59	M	1	Restricted trauma-induced dystonia	Trauma (incidental cavernoma)
	S5.	34	M	0	Secondary generalized	Blood asphyxia
	S6.	19	M	1	Secondary generalized hemidystonia	Basal ganglia dysfunction (dissection of ICA on the MRA), LH stroke
	S7.	47	M	0	Secondary generalized hemidystonia	Early cerebral trauma and TBM
	S8.	52	M	0	Right torticollis and hemiballism	Stroke following major LH thalamic hemorrhage
	S9.	34	F	0	Secondary generalized, right limb spasticity	Severe anoxia at birth
	S10.	27	M	0	Secondary generalized	Severe hypoxia at birth
	S11.	21	M	1	Secondary generalized hemidystonia	TBM at eight months

One patient with generalized secondary dystonia to anoxia at birth presented with severe motor and cognitive difficulties of a degree to preclude formal assessment. Three of the patients were assessed both pre- and post-DBS. Of the 21 patients who were assessed, three were only able to perform on selected tests due to the extensive limitations of their dystonia. For the remaining 18 patients, there were only very occasional tests that could not be administered due to limitations of their dystonia.

Procedure 

All patients underwent neuropsychological testing using our DBS protocol and neuropsychiatric assessment as part of their routine pre-operative evaluation to assess suitability for DBS. Five main cognitive domains were assessed: (i) intellectual, (ii) memory, (iii) language (iii) perception and (iv) executive functions. Neuropsychiatric examination consisted of one interview session and, with patient permission, a corroborative history provided by a spouse or primary caregiver.

Results 

Comparisons between Primary and Secondary Dystonia Patients and Normative Data on Neuropsychological Tests 

Neuropsychological performance data are summarized in Table 2. Normative comparisons showed that Brixton task performance was significantly impaired in the primary dystonia patient group (M = 5.36, SD = 3.38). There was no corresponding impairment on the Digit Span task of working memory (M = 8.1, SD = 3.03). Brixton task performance was preserved in the secondary dystonia patient group. The difference between group means was found to be statistically significant: t (18) = 2.317, p = .033. Performance was preserved on all remaining neuropsychological measures and there were no significant differences between mean test scores in primary and secondary dystonia patients (all p values > .05). All 16 patients who provided sufficient data for premorbid IQ estimations obtained full-scale IQ (FSIQ) scores within their optimal range.

**Table 2:** Mean and standard deviation scores for neuropsychological tests in patients with primary or secondary dystonia. p value relates to comparisons with normative data (see text for details). ^¥^ Bonferroni corrected for multiple comparisons. ^*^**p< 0.05. ^**^ p< 0.005. ^a^* *Number of patients scoring ≥ 2 SD below the test’s normative mean (i.e., age scaled scores > 5). ^1^ WAIS-III: VIQ, PIQ and FSIQ normative mean for each = 100 and 2 SD = 30 IQ points. ^2^ Age corrected scaled scores frequently used for neuropsychological tests have a range of 1-20 with a mean = 10 and 2 SD = 6 used for subtest of the WAIS-III, memory tests, verbal fluency tests, GNT, incomplete letters, object decision and Brixton tests as need to be age corrected. FSIQ: Full-scale IQ; VIQ: Verbal IQ; PIQ: Performance IQ; WAIS: Wechsler adult intelligence scale.

	n	Mean	SD	p-value^¥^	N patients impaired (scores ≥ 2 SD ^a^)
Primary dystonia					
Estimated Premorbid IQ Intellectual tests	8	91.88	12.07	0.489	1
FSIQ^1^	10	88.4	14.45	0.285	1
VIQ^1^	10	88	13.18	0.262	1
PIQ^1^	11	90.46	17.45	0.373	1
Memory Tests					
Recognition					
Forced-choice words^2^	11	9.27	2.27	0.71	0
Forced-choice faces^2^	11	8.36	4.52	0.477	2
Recall					
Story recall immediate^2^	10	7	3.16	0.178	5
Story recall delayed^2^	10	7.2	3.49	0.218	3
List-learning^2^	10	7.2	3.43	0.216	4
Language Tests					
Graded naming test	11	7.09	2.63	0.156	4
Perceptual Tests					
Incomplete letters	11	14.46	4.16	0.056	0
Object decision	11	8	2.57	0.322	3
Executive Tests					
Brixton	11	5.36	3.38	0.037^*^	6
Hayling	10	7.7	2.5	0.278	2
Letter fluency	10	9.8	4.96	0.936	3
Category fluency	10	7.6	3.6	0.292	3
Secondary dystonia					
Estimated Premorbid IQ Intellectual tests	8	91	13.44	0.452	1
FSIQ^1^	9	85.33	10.83	0.187	1
VIQ^1^	8	85	10.46	0.2031	1
PIQ^1^	9	84	11.95	0.157	1
Memory Tests					
Recognition					
Forced-choice words^2^	10	7.9	4.36	0.382	3
Forced-choice faces^2^	10	9.1	5.07	0.721	3
Recall					
Story recall immediate^2^	7	7.57	1.62	0.321	1
Story recall delayed^2^	7	6.86	2.48	0.225	2
List-learning^2^	8	5.75	2.96	0.094	4
Language Tests					
Graded naming test	8	7	4.41	0.274	4
Perceptual Tests					
Incomplete letters	10	14	4.22	0.102	0
Object decision	9	8.56	4.48	0.572	1
Executive Tests					
Brixton	9	9.11	3.86	0.713	1
Hayling	7	7.71	2.75	0.377	2
Verbal fluency	7	7.14	4.18	0.321	2
Category fluency	7	7.71	4.35	0.43	1

Comparison of neuropsychological assessment results suggested superior verbal memory in the primary dystonia group. Relative to the secondary dystonia group, performance means were higher for the forced-choice paradigm for words (M = 9.27, SD = 2.27 vs. M = 7.9, SD = 4.36), List Learning task (M = 7.2, SD = 3.43 vs. M = 5.75, SD = 2.96) and phonemic verbal fluency (M = 9.8, SD = 4.96 vs. M = 7.14, SD = 4.18). A greater number of individual impairments were observed in the secondary dystonia group for all measures of verbal memory. Still, no significant differences were found between group means on these tests.

Comparisons of Frequency of Individual Cognitive Deficits According to Normative Test Data 

Variability of cognitive performance amongst patients within each dystonia group presents the possibility that there is weak regression of individual test scores to the mean. Therefore, individual impairments were determined relative to normative test data for each patient. Impaired intellectual functions were entirely consistent with below average estimated premorbid levels of functioning in two patients. No intellectual impairments were found on composite IQ measures for the remaining 14 patients who provided premorbid IQ estimates. With regards to memory, considerably more primary dystonia patients demonstrated impairment on the Immediate Story Recall task. However, failure on the forced-choice paradigm for words was selective to the secondary dystonia group. On average, List Learning performance was relatively poor amongst secondary dystonia patients. There was a similar distribution of language and perception deficits in the two groups. However, Object Decision task failure was more common in the primary dystonia group.

Executive dysfunction was more widespread amongst primary dystonia patients and appeared to be predicted by Brixton task failure. Only one patient (P6) demonstrated Brixton task impairment without a more generalized compromise of executive functions. Deficits were less prevalent and more widely distributed across executive tasks in the secondary dystonia group. Of the five secondary dystonia patients that demonstrated executive deficits, only one (S4) was impaired on more than one executive task: the Brixton task and the Hayling test. The remaining four patients showed deficits that were specific to the Hayling test or one of the vocabulary assessment scale (VAS) fluency tasks. Therefore, generalized executive impairment suggested by > one task deficit was entirely predicted by Brixton task failure in both patient groups.

**Figure 1:**
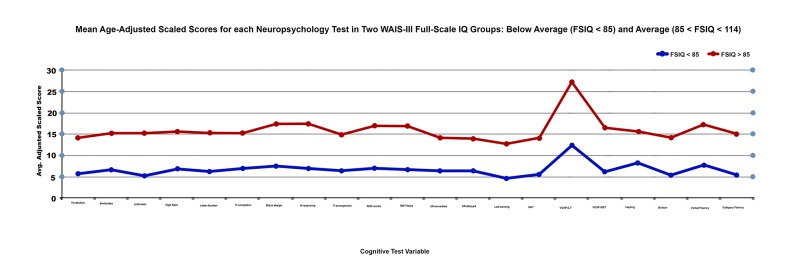
Mean neuropsychological test scores in average and below average FSIQ groups. FSIQ: Full scale intelligence quotient

To assess the relationship between FSIQ and cognitive performance, patients were classified as below-average (BA) FSIQ if they scored ≥ 1 SD below the normative mean and average (A) if they scored within the normal range of 85-114 IQ points. Figure 1 compares mean performance scores in the BA (n = 8) and A (n = 11) FSIQ groups across all neuropsychological tests. 42.1% patients in the BA group demonstrated poor performance relative to the 57.9% in the A group across all measures. However, no significant differences were found between group means. Cognitive performance in the subpopulation of BA FSIQ patients was well correlated with that of patients with A FSIQ in all test domains. FSIQ was not impaired relative to estimated premorbid IQ in the BA FSIQ group (mFSIQBA = 76.25 vs. mPreIQBA = 81.71) or the A FSIQ group (mFSIQA = 94.73 vs. mPreIQA = 99) (all p values > .05). Therefore, there was no indication of intellectual decline in either group.

Prevalence of Psychiatric Illness in Primary and Secondary Dystonia Patients 

Clinical psychiatric illness occurred in four primary dystonia patients with an estimated prevalence of 36.36%. There was no indication of current mental illness amongst all primary dystonia patients. Past history of major depressive disorder (MDD) was consistently reported amongst clinical psychiatric histories and all patients had been treated with antidepressant medications. Of the four reported cases of MDD, two included suicidal ideations, two included comorbid anxiety disorders, two included substance abuse and one included a forensic history. All four patients reported low mood relating to the disabling effects of their dystonia.

Amongst secondary dystonia patients, clinical psychiatric illness occurred in four patients with an estimated prevalence of 40%. There was considerable variability amongst secondary dystonia patients with clinical psychiatric histories. Cases involved: schizophrenic psychosis (S1), anxiety with rage and comorbid mood dysregulation (S7), MDD with suicidal ideations (S8), and PTSD with anxiety and a history of physical abuse (S9). Past and current anxiety reported in one patient (S2) suggested worsening over time. However, symptoms were deemed to derive from the disabling effects of the patients’ cerebral palsy and secondary dystonia. One patient (S1) was continued on neuroleptic medication for control of his schizophrenia but had not experienced a psychotic episode for 15 years prior to preoperative assessment.

Expectations and Concerns Relating to DBS Procedure 

All patients predicted that the DBS surgery would be successful with a mean expected motor symptom improvement of 43.21% agreed between physicians and patients. Expectations of surgery for motor features of dystonia were high amongst primary dystonia patients relative to secondary dystonia patients (45.38% vs. 39.69%). However, this did not reach statistical significance (p > .05). In the primary dystonia group (n = 11) three patients were concerned about the neuropsychiatric implications of DBS and one patient was concerned about short-term memory decline. In the secondary dystonia group (n = 10) three patients were concerned about the neuropsychiatric implications of DBS and two patients were concerned about cognitive functions related to short-term memory, attention and concentration. In both groups, neuropsychiatric concerns related to possible exacerbation of low mood, frustration or anxiety were raised should the disabling motor difficulties persist and continue to limit social interactions after surgery.

Pre- and Post-DBS Comparisons

**Figure 2:**
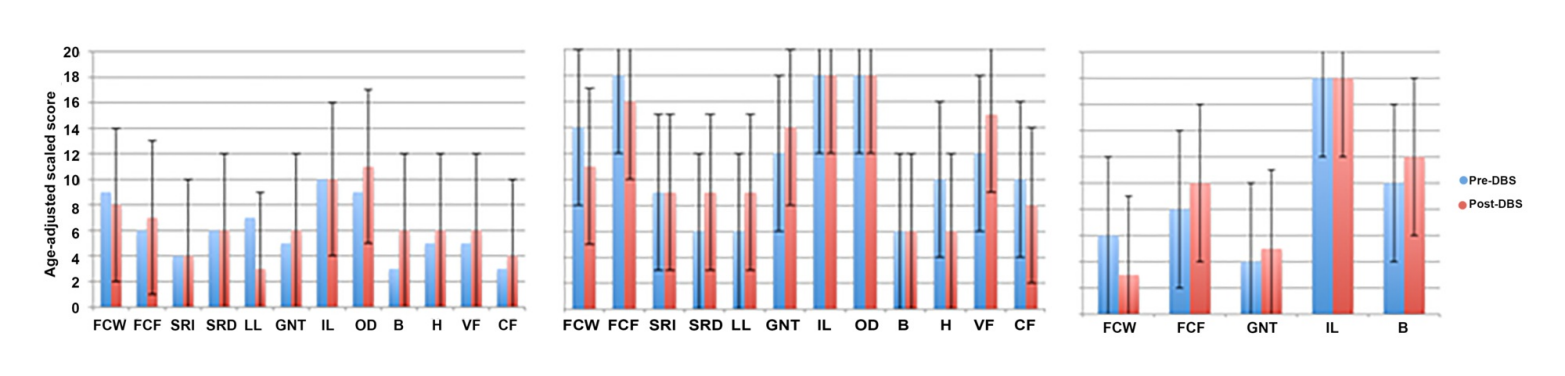
Clustered bar graphs to show age adjusted scaled scores for memory, language, perception and executive functions at both test phases. Error bars signify the criterion for significant differences in cognitive functions: ≥6 scaled score points (left: P1, centre: S1, right: S2).

Neuropsychological testing (Table 3) provides pre-operative and post-operative neuropsychological test performance data for all three patients including statistically significant impairments from normative comparisons. The data is represented graphically in Figure 2 to elucidate possible patterns of altered function in each patient that may be of interest despite failing to satisfy our stringent criteria for difference score significance.

**Table 3:** Raw IQ scores and age adjusted scaled scores for all remaining neuropsychological tests in patients P1, S1 and S2 at the time of pre-operative assessment and six months following GPi-DBS surgery. Pre-DBS= Pre-operative neuropsychological test data obtained at the time of clinical assessment; Post-DBS= Post-operative neuropsychological test data obtained six months following GPi-DBS surgery. Premorbid IQ estimates were computed pre-operatively. *D* = Difference score calculated by Post-DBS score minus Pre-DBS score. FAS: Verbal fluency test where patients form as many words as possible with letters F,A,S. ^a^* *P1*. *= Primary dystonia patient no. 1. ^b^* *S1*. *= Secondary dystonia patient no. 1. ^c^* *S2*. *= Secondary dystonia patient no. 2. DBS: Deep brain stimulation; VIQ: Verbal IQ; PIQ: Performance IQ; FSIQ: Full-scale IQ; GPi: Globus pallidus internus.

	P1.^ a^			S1.^ b^			S2.^ c^		
	Pre-DBS	Post-DBS	D	Pre-DBS	Post-DBS	D	Pre-DBS	Post-DBS	D
Neuropsychological test									
Premorbid IQ	70^*^			110			90		
VIQ	64^*^	65^*^	1	91	99	8	-	-	
PIQ	69^*^	81	12	86	87	1	-	-	
FSIQ	67^*^	70^*^	3	88	94	6	97	94	-3
Forced choice - Words	9	8	-1	14	11	-3	6	3^*^	-3
Forced choice - Faces	6	7	1	18	16	-2	8	10	2
Story recall - Immediate	4^*^	4^*^	0	9	9	0	-	-	
Story recall - Delayed	6	6	0	6	9	3	-	-	
List learning	7	3^*^	-4	6	9	3	-	-	
Graded naming	5^*^	6	1	12	14	2	4^*^	5^*^	1
Incomplete letters	10	10	0	18	18	0	18	18	0
Object decision	9	11	2	18	18	0	-	-	
Brixton	3^*^	6	3	6	6	0	10	12	2
Hayling	5^*^	6	1	10	6	-4	-	-	
FAS verbal fluency	5^*^	6	1	12	15	3	-	-	
Category fluency	3^*^	4^*^	1	10	8	-2	-	-	

Visual inspection of the data provides no indication of altered intellectual functioning following DBS surgery relative to performance at the time of initial assessment. There are no significant differences between IQ performance scores at either stage of assessment and the estimated optimal levels of functioning in either patient. Intellectual functions across assessment phases for patient S2 as verbal IQ (VIQ) and performance IQ (PIQ) data were missing. However, FSIQ performance was similar in both test phases (difference score of three IQ points equivalent to 0.2 SD points) and was proximal to the patients’ estimated premorbid IQ. Therefore the available data provide no indication of intellectual decline prior to or following DBS. Comparisons between pre-operative and post-operative performance on all remaining neuropsychological tests reveals no significant deterioration of cognitive functioning.

## Discussion

Frequency and phenotypes of cognitive deficits

Deficits in cognitive functions were commonly identified in this group of dystonia patients referred for consideration of DBS with 80.95% of patients found to have at least one recordable cognitive deficit. The incidence is likely to have been high due to the panoptic battery of neuropsychometric testing employed.

Regarding the phenotypes of cognitive deficits, the neuropsychological profiles showed below average intellectual functions, reaching statistical significance in only two patients. As was expected, low-level intellectual functions predicted a high frequency of deficits across the main cognitive test domains.

There was preservation of language and perceptual functions as expected. Although mean test scores failed to reach statistical significance, individual impairments were commonly detected on tests of memory function, particularly relating to auditory and visual learning. With respect to executive functions, performance on the Brixton test of spatial anticipation was selectively impaired in the primary dystonia patients. Brixton task failure was a reliable predictor of additional executive deficits in primary dystonia patients, whereas deficits were less frequent and more sparsely distributed across executive tests in the secondary dystonia patients. Brixton task performance was significantly impaired in primary relative to secondary dystonia patients, representing the only clinically significant difference in cognitive functions between the two groups.

Given the heterogeneity of primary dystonia syndromes in our sample, it is unlikely that Brixton task failure was the product of common pathogenic processes in the affected patients. Fifty percent of pre-operative Brixton test deficits were attributed to patients with DYT-1 dystonia. Manifesting and non-manifesting carriers of the DYT-1 mutation demonstrate a significant sequence-learning deficit and concomitant increases in cerebellar activation on task performance [12]. As this is not attributed to interference from dystonic symptoms, impaired sequence learning is recognized as a specific feature of the DYT-1 genotype [13]. Given that all DYT-1 dystonia patients in the current study exhibited impaired induction of rules from sequences of stimuli on the Brixton test, increased cerebellar activation may represent a common pathophysiological substrate for Brixton test failure and the sequence-learning deficit in DYT-1 dystonia.

Absence of functional cognitive decline following DBS

We found no indication of cognitive decline following DBS in the three patients that were assessed pre- and post-operatively, finding that has been replicated across other studies and various methods of cognitive assessment wherein DBS has successfully treated dystonic symptoms [14]. Mild performance fluctuations were bidirectional and insignificant and postoperative outcomes were not predicted by baseline cognitive evaluations. As was expected, there was considerable variation amongst patients and no notable differences emerged between primary and secondary dystonia patients in relation to DBS. Furthermore, baseline cognitive deficits were not exacerbated post-operatively, at six months post-operatively, suggesting the safety of DBS for patients with already low-level cognitive functions.

Functionality and integrity of depressed mood in dystonia

The majority of the existing literature has focused on psychiatric aspects of primary dystonia, consistently implicating depression as the most prevalent comorbidity when compared with other psychiatric health problems [15]. With one notable exception [16], the severity of depression in dystonia patients has not been correlated with age, botulin toxin treatment duration, or the severity or duration of the dystonia [15], suggesting that it may be a primary feature of pathophysiology in pure dystonias. The relative breadth of psychiatric morbidity in secondary dystonia patients implies a functional dichotomy between depression and other psychiatric illnesses including anxiety in the etiology of primary dystonia. The current study does not elucidate the temporal relations between psychiatric disturbances and motor manifestations of dystonia. However, our collective findings seem to support the view that major depressive illness may be an inherent component of pathophysiology in dystonia, in line with clinical observations of depression in dystonia patients and identification of shared cortical-limbic-striatal circuitry dysfunctions.

Sensitivity of psychiatric status to DBS

DBS did not appear to produce any adverse psychiatric effects in the three patients that were assessed postoperatively. Current findings are consistent with previous studies of patients with similar pre-operative neuropsychiatric profiles to those reported here. Mood remained stable in our sample of non-depressed dystonia patients [17] and there were no clinically relevant exacerbations of anxiety [18]. As was expected, incidence of mild post-operative anxiety was confounded by withdrawal of anxiety reducing medications and effects of emotional stress. Given the main clinical concern of suicidality risk following GPi-DBS in dystonia, the absence of even the most minor decrements in mood in our sample of patients may provide preliminary reassurance for patients with treatment-resistant dystonias. However, the delayed emergence of anxiety and its detrimental effects on motor manifestations in P1 emphasize the therapeutic value of routine neuropsychiatric examinations in DBS. Furthermore, persistence of anxious tendencies in S2 supports long-term personality attributes related to emotional processing difficulties, such as less openness and perfectionism, as enduring predispositions in dystonia that may not be amenable to change following DBS [19].

Limitations

Clinical interpretation of current findings is hampered by various methodological limitations. The small size and selectivity of our sample are clear limitations of the study. Furthermore, the inherent heterogeneity of clinical manifestations in our sample limits the power of formal statistical comparisons, as there is a problem of regression to the mean. Regarding cognitive functions, clinical interpretation of findings in light of the available evidence is complicated by the multiplicity of neuropsychological assessment tools used and limitations to age-correcting methodology as cognitive measures become less fine-grained. Lastly, whilst neuropsychiatric interviews provide a relatively thorough reflection of the considerably varied individual psychiatric profiles, the qualitative nature of the data provides a low-resolution image of underlying trends. Future studies may make use of formal assessments in evaluating subjective mood and anxiety states as well as emotional and social ratings in order to provide more informative accounts of baseline psychiatric aspects and measurable changes following DBS.

## Conclusions

Common detection of cognitive deficits and psychiatric disturbances in patients disparage a purely “motor” definition of dystonia. Given the inherent heterogeneity of dystonia syndromes and their associated effects, considerable variability in the non-motor features manifestations is to be expected. Moreover, the absence of shared cognitive deficits amongst dystonias that fall into primary or secondary etiological classifications is unsurprising. The frequency of individual impairments suggests that difficulties associated with dystonia are likely to be of clinical relevance to cognitive functions in the majority of patients. In particular, current findings suggest that executive difficulties related to inductive processes and spatial learning may be a common in primary dystonias. Psychiatric disturbances demand recognition as a central aspect of dystonia as they contribute to overall disease burden, poor quality of life and exacerbated motor disabilities. The functionality of psychiatric abnormalities in the etiology of dystonia remains ultimately unresolved, but the available evidence provides overwhelming suggestion that vulnerability to depression is inherent to the dystonia phenotype. Determinants of psychiatric disturbance such as stress, low self-esteem and social isolation should be avoided so as not to aggravate motor manifestations of dystonia. Moreover, possible risk factors for mental health problems should be acknowledged in DBS treatment procedures. Advances in understanding the clinical features of dystonia may inform pre-operative assessment methods for candidate selection, lead to identification of novel targets and parameters for stimulation, and aid post-operative treatment plans in DBS so that therapeutic benefit can be optimized without risk to cognitive functions or psychiatric wellbeing.

## References

[1] Balas M, Peretz C, Badarny S, Scott RB, Giladi N (2006). Neuropsychological profile of DYT1 dystonia. Mov Disord.

[2] Bugalho P, Corrêa B, Guimarães J, Xavier M (2008). Set‐shifting and behavioral dysfunction in primary focal dystonia. Mov Disord.

[3] Scott RB, Gregory R, Wilson J (2003). Executive cognitive deficits in primary dystonia. Mov Disord.

[4] Pillon B, Ardouin C, Dujardin K (2006). Preservation of cognitive function in dystonia treated by pallidal stimulation. Neurology.

[5] Bressman SB, Saunders‐Pullman R (2013). Primary dystonia: moribund or viable. Mov Disord.

[6] Freeman K, Gregory A, Turner A, Blasco P, Hogarth P, Hayflick S (2007). Intellectual and adaptive behaviour functioning in pantothenate kinase‐associated neurodegeneration. J Intellect Disabil Res.

[7] Heiman GA, Ottman R, Saunders-Pullman RJ, Ozelius LJ, Risch NJ, Bressman SB (2004). Increased risk for recurrent major depression in DYT1 dystonia mutation carriers. Neurology.

[8] Jankovic J (2006). Treatment of dystonia. Lancet Neurol.

[9] Vidailhet M, Vercueil L, Houeto J-L (2005). Bilateral deep-brain stimulation of the globus pallidus in primary generalized dystonia. N Engl J Med.

[10] Moro E, Gross RE, Krauss JK (2013). What's new in surgical treatment for dystonia?. Mov Disord.

[11] Foncke EM, Schuurman PR, Speelman JD (2006). Suicide after deep brain stimulation of the internal globus pallidus for dystonia. Neurology.

[12] Carbon M, Argyelan M, Ghilardi MF, Mattis P, Dhawan V, Bressman S, Eidelberg D (2011). Impaired sequence learning in dystonia mutation carriers: a genotypic effect. Brain.

[13] Ghilardi MF, Carbon M, Silvestri G (2003). Impaired sequence learning in carriers of the DYT1 dystonia mutation. Ann Neurol.

[14] Gruber D, Trottenberg T, Kivi A (2009). Long-term effects of pallidal deep brain stimulation in tardive dystonia. Neurology.

[15] Fabbrini G, Berardelli I, Moretti G (2010). Psychiatric disorders in adult‐onset focal dystonia: a case‐control study. Mov Disord.

[16] Gundel H, Busch R, Ceballos-Baumann A, Seifert E (2007). Psychiatric comorbidity in patients with spasmodic dysphonia: a controlled study. J Neurol Neurosurg Psychiatry.

[17] Vidailhet M, Vercueil L, Houeto J-L (2007). Bilateral, pallidal, deep-brain stimulation in primary generalised dystonia: a prospective 3 year follow-up study. Lancet Neurol.

[18] Valldeoriola F, Regidor I, Minguez-Castellanos A (2010). Efficacy and safety of pallidal stimulation in primary dystonia: results of the Spanish multicentric study. J Neurol Neurosurg Psychiatry.

[19] Lencer R, Steinlechner S, Stahlberg J (2009). Primary focal dystonia: evidence for distinct neuropsychiatric and personality profiles. J Neurol Neurosurg Psychiatry.

